# Therapeutic Efficacy of Silymarin, Vitamin E, and Essential Phospholipid Combination Therapy on Hepatic Steatosis, Fibrosis, and Metabolic Parameters in MASLD Patients: A Prospective Clinical Study

**DOI:** 10.3390/ijms26125427

**Published:** 2025-06-06

**Authors:** Dan-Ionuț Gheonea, Cristina Tocia, Victor-Mihai Sacerdoțianu, Alexandra-Georgiana Bocioagă, Irina-Paula Doica, Nicolae Cătălin Manea, Adina Turcu-Știolică, Carmen-Nicoleta Oancea, Eugen Dumitru

**Affiliations:** 1 Research Center of Gastroenterology and Hepatology, University of Medicine and Pharmacy Craiova, 200349 Craiova, Romania; dan.gheonea@umfcv.ro (D.-I.G.); alexandra.bocioaga@umfcv.ro (A.-G.B.); irina.doica@umfcv.ro (I.-P.D.); 2 Gastroenterology Department, “Sf. Apostol Andrei” Clinical Emergency County Hospital, 145 Tomis Blvd., 900591 Constanta, Romania; cristina.tocia@yahoo.com (C.T.); eugen.dumitru@yahoo.com (E.D.); 3 Faculty of Medicine, Ovidius University of Constanta, 1 Universitatii Street, 900470 Constanta, Romania; 4 Department of Biochemistry, University of Medicine and Pharmacy of Craiova, 200349 Craiova, Romania; carmen.oancea@umfcv.ro; 5 Department of Medical Informatics and Biostatistics, University of Medicine and Pharmacy Craiova, 200349 Craiova, Romania; catalin.manea@umfcv.ro; 6 Biostatistics Department, Faculty of Pharmacy, University of Medicine and Pharmacy Craiova, Petru Rares Street No 2-4, 200349 Craiova, Romania; adina.turcu@umfcv.ro; 7 Center for Research and Development of the Morphological and Genetic Studies of Malignant Pathology, Ovidius University of Constanta, 145 Tomis Blvd., 900591 Constanta, Romania; 8 Academy of Romanian Scientist, 3 Ilfov Street, 050044 Bucharest, Romania

**Keywords:** MASLD, silymarin, VCTE

## Abstract

Metabolic dysfunction-associated steatotic liver disease (MASLD) is the most prevalent chronic liver disease globally, and current estimates indicate an increase in incidence and prevalence in the general population. The design of the prospective study was to evaluate the response of patients with MASLD to an original formula consisting of silymarin, vitamin E, and essential phospholipids. In total, 200 patients were initially enrolled in the study and a total of 190 who participated in all four visits were included in our analysis. During the visits, liver function tests, lipid profiles, blood glucose level, fibrosis, and steatosis values and grades were assessed. From baseline, visit 0, to month 6th, visit III, a statistically significant difference (*p*-value < 0.0001) was observed in the reduction in ALT, AST, GGT, ALP, TG, total cholesterol, and blood glucose levels. There was a significant decrease in the fibrosis value from the first visit to the last visit (*p* = 0.002). Even though administered separately, silymarin, essential phospholipids, and vitamin E have established their efficacy in MASLD, this study demonstrates that their combination produces an indubitable effect on liver steatosis, even in a short cure of 6 months, and it can be proposed due to it having no adverse effects on patients with MASLD.

## 1. Introduction

Metabolic dysfunction-associated steatotic liver disease (MASLD), the most prevalent chronic liver disease globally, will become one of the most important causes of cirrhosis due to the increased number of cases. Metabolic dysfunction-associated steatohepatitis (MASH) is a disease included in the MASLD spectrum, which is associated with inflammation in hepatic parenchyma [1,2].

Diagnosis of MASLD is made using transabdominal ultrasound that showed hyperechoic liver parenchyma with certain signs associated with the grade of steatosis. Even transabdominal ultrasound can be used for a severity classification, and other techniques can highlight an objective measurement.

Magnetic resonance elastography (MRE) is one of the techniques that can be used to assess the patient with hepatic steatosis, but the cost and time consumption are disadvantages. Also, adults with morbid obesity may not be suitable for this assessment [3,4,5].

Vibration-controlled transient elastography (VCTE) with controlled attenuation parameter (CAP) and liver stiffness measurement (LSM) is a suitable tool that can be used for both the diagnosis and quantification of steatosis and fibrosis [6,7].

The role of oxidative stress in MASLD was analyzed in multiple studies. It was demonstrated that fat accumulation in hepatocytes leads to the production of reactive oxygen species (ROS) and inflammatory cytokines. Oxidative stress stimulates hepatic stellate cells, Kupffer cells, and hepatocytes and can induce inflammatory changes, switching from simple steatosis to MASH. All these changes are reversible when a diet or an antioxidant agent is initiated. Unfortunately, when the process is progressive, the transformation to MASH is very probable, and fibrosis occurs [8,9,10,11].

Actual European guidelines recommend a Mediterranean or similar diet, as well as weight loss to improve liver injury for patients with MASLD. Physical activity and exercise have also been recommended for these patients, especially for the cardiometabolic effects. There are multiple pharmacological therapies recommended for MASLD that include silymarin, essential phospholipids, vitamin E, organic selenium, and/or alpha lipoic acid, as well as a new product that is unfortunately unavailable in our country (Romania), a thyroid hormone receptor agonist of the beta subtype named resmetirom [1,4,12,13].

Silymarin is one of the most used drugs for the treatment of steatotic liver disease due to its anti-inflammatory, antioxidant, and anti-fibrotic activity that is associated with beneficial results and a good safety profile [14,15,16].

The phosphatidylcholine-to-phosphatidylethanolamine ratio in hepatocytes is proposed as a determinant key of MAFLD, and treatment with essential phospholipids is argued. Polyenylphosphatidylcholine is one of the essential phospholipids and a major component of biological membranes. When a low level of phosphatidylcholine occurs, the liver decreases the secretion of very low-density lipoprotein cholesterol with the accumulation of lipids in hepatocytes, and de novo lipogenesis also begins [17].

Vitamin E has demonstrated an antisclerotic effect and was also found to reduce total serum cholesterol, LDL-cholesterol, and triglycerides (TGs) [18,19,20].

Since every substance, silymarin, essential phospholipid, and vitamin E has shown efficacy on liver steatosis but with various results, the hypothesis that combination therapy may be a better solution for patients with MASLD than a single therapy with any of these substances was proposed.

The aim of this study was to highlight the effect of the abovementioned formula consisting of silymarin, vitamin E, and essential phospholipids on patients with MASLD by evaluating liver function tests, lipid profiles, blood glucose levels, values, and degrees of fibrosis and steatosis.

## 2. Results

**Figure 1:**
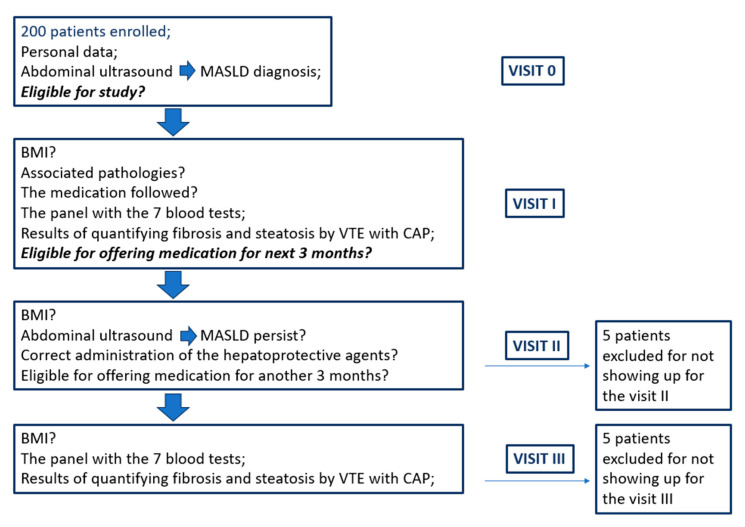
Flowchart of the patients included in the study.

The study initially enrolled 200 patients, with 190 completing all four visits, and they included in the final analysis. Five participants attended only visit 0 and visit I, while another five did not complete the last visit, visit III. One patient of the last five that did not complete visit III discontinued medication due to lower limb edema, which was initially self-reported as a potential adverse event. Subsequent cardiology evaluation revealed cardiac decompensation as the underlying cause (Figure 1).

The demographic and clinical characteristics of the participants are summarized in Table 1. The mean ± SD age of the cohort was 47.66 ± 10.43 years, the majority being male, representing 52.6% of the cohort. A total of 26.3% of participants reported no alcohol consumption, whereas 57.4% reported occasional alcohol consumption and 13.2% reported frequent alcohol consumption. Four comorbidities were assessed: 35.3% of the cohort had a diagnosis of type 2 diabetes, 40.5% of participants were diagnosed with hypertension, nearly half of the cohort (49.5%) had dyslipidemia, and 15.3% of participants were diagnosed with hypothyroidism.

The mean LSM was 6.14 ± 6.68 kPa, with a median value of 5.4 kPa. The majority of participants (82.1%) had fibrosis grades F0–F1. The mean ± SD CAP score was 296.05 ± 36.82 dB/m, with a median value of 293 dB/m. In total, 22.1% of participants had steatosis grade S1, whereas 38.9% had steatosis grade S2 and 38.9% had steatosis grade S3. The data from each visit and the statistical differences between them are presented in Table 2.

### 2.1. BMI

**Figure 2:**
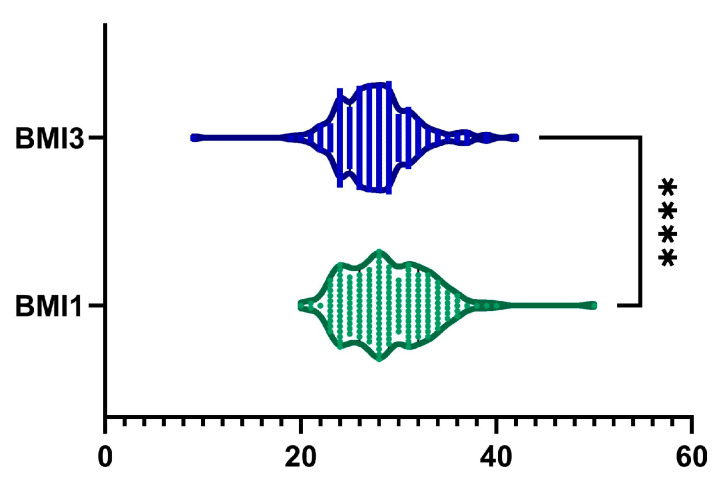
A reduction in BMI was observed after 6 months of treatment (BMI1, visit I vs. BMI3, visit III). ****, *p* < 0.0001.

The BMI values ranged from 20 kg/m^2^ to 50 kg/m^2^. The mean (±SD) BMI at visit I was 28.97 ± 4.24 kg/m^2^, which decreased to 28.03 ± 3.64 kg/m^2^ at visit II and further to 27.65 ± 3.89 kg/m^2^ at visit III. A statistically significant reduction in BMI was observed after 6 months of treatment (*p*-value < 0.0001, Figure 2). The overall reduction in BMI from the first visit to the last visit was 4.1 ± 8.4%. Among the cohort, 114 patients (60%) exhibited a reduction in BMI, while 46 patients (24.21%) experienced weight gain. Among male patients, we observed a statistically significant decreased BMI from 16.32 ± 15.28 kg/m^2^ to 15.61 ± 14.62 kg/m^2^ (*p* < 0.0001). In contrast, among female patients, the reduction in BMI from 13.15 ± 15.09 kg/m^2^ to 12.88 ± 14.8 kg/m^2^ was not statistically significant (*p* = 0.06).

### 2.2. ALT

**Figure 3:**
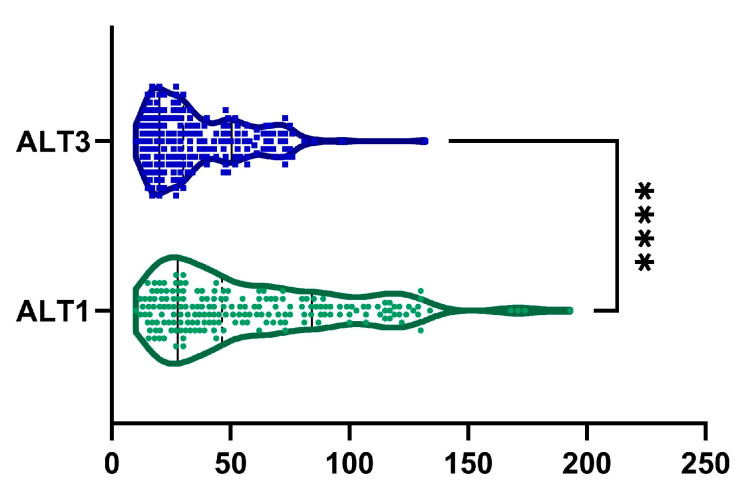
Reduction in ALT was observed after 6 months of treatment (ALT1, visit I vs. ALT3, visit III). ****, *p* < 0.0001.

An 18.72 ± 53.86% reduction in ALT levels was observed from baseline to month 6, with a statistically significant difference, as shown in Figure 3 (*p*-value < 0.0001). Furthermore, 144 patients (75.8%) demonstrated a reduction in ALT levels. Among male patients, we observed a statistically significant decreased ALT from 57.04 ± 37.9 U/L to 37.79 ± 20.07 U/L (*p* < 0.0001). Similarly, among female patients, ALT decreased statistically from 60.44 ± 39.16 U/L to 35.82 ± 21.39 U/L (*p* < 0.0001).

### 2.3. AST

**Figure 4:**
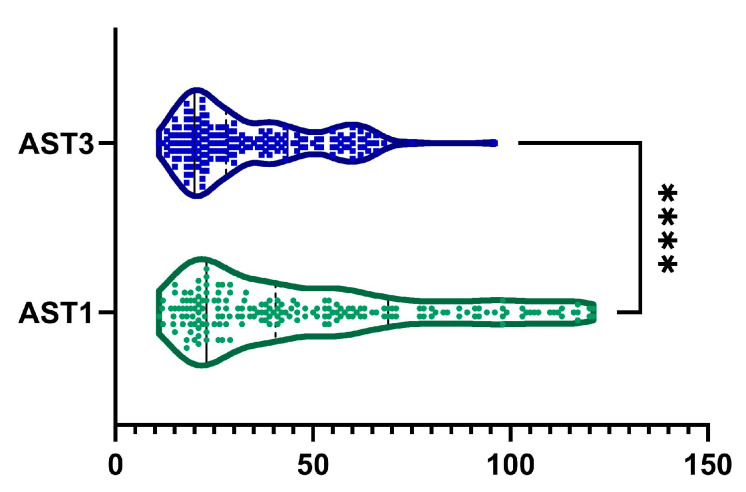
Reduction in AST was observed after 6 months of treatment (AST1, visit I vs. AST3, visit III). ****, *p* < 0.0001.

A 15.72 ± 43.54% reduction in AST levels was observed from baseline to month 6, with a statistically significant difference, as illustrated in Figure 4 (*p*-value < 0.0001). Additionally, 129 patients (67.9%) exhibited a reduction in AST levels. Among male patients, we observed a statistically significant decreased AST from 48.31 ± 29.23 U/L to 32.06 ± 15.08 U/L (*p* < 0.0001). Similarly, among female patients, AST decreased statistically from 50.82 ± 33.11 U/L to 34.89 ± 18.15 U/L (*p* < 0.0001).

### 2.4. GGT

**Figure 5:**
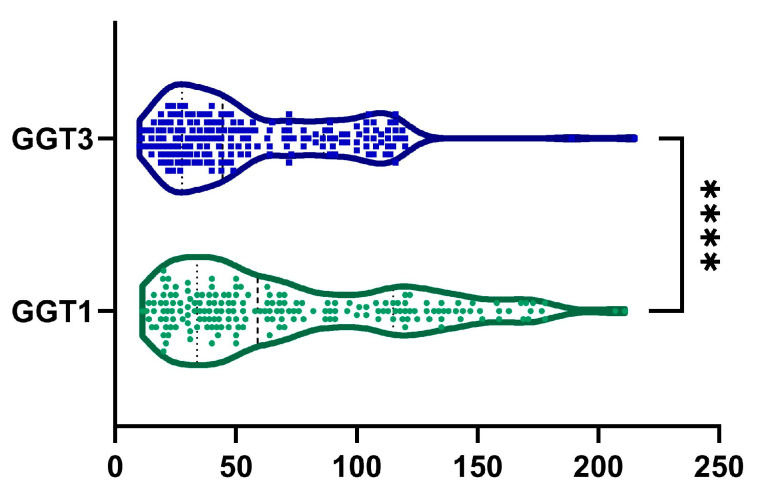
Reduction in GGT was observed after 6 months of treatment (GGT1, visit I vs. GGT3, visit III). ****, *p* < 0.0001.

A 6.39 ± 76.65% reduction in GGT levels was observed from baseline to month 6, with a statistically significant difference, as illustrated in Figure 5 (*p*-value < 0.0001). Additionally, 134 patients (70.5%) exhibited a reduction in GGT levels. Among male patients, we observed a statistically significant decreased GGT from 73.42 ± 47.57 U/L to 56.16 ± 34.23 U/L (*p* < 0.0001). Similarly, among female patients, GGT decreased statistically from 73.52 ± 49.86 U/L to 57.87 ± 37.96 U/L (*p* < 0.0001).

### 2.5. ALP

**Figure 6:**
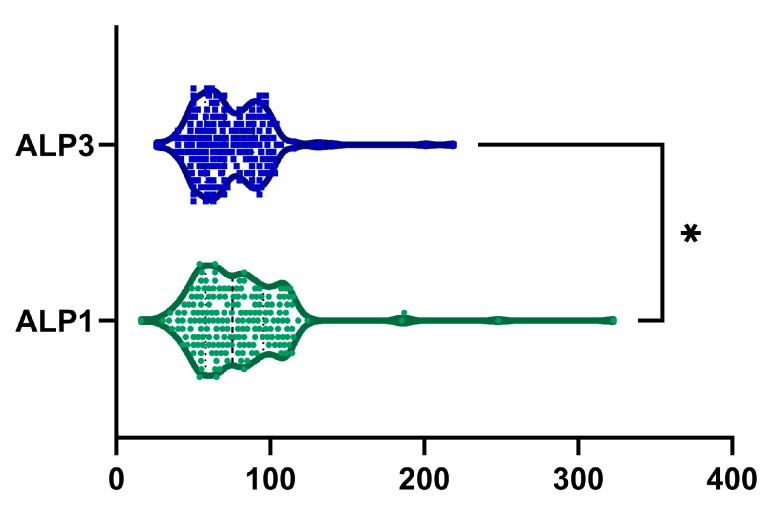
Reduction in ALP was observed after 6 months of treatment (ALP1, visit I vs. ALP3, visit III). *, *p* < 0.05.

An average 2.1 ± 42.02% reduction in ALP levels was observed from baseline to month 6, with a statistically significant difference, as illustrated in Figure 6 (*p*-value = 0.022). Furthermore, 108 patients (56.8%) demonstrated a reduction in ALP levels. Among male patients, we observed no statistically significant decrease in ALP levels from 74.42 ± 28.49 U/L to 72.87 ± 25.5 U/L (*p* = 0.526). In contrast, among female patients, ALP levels decreased significantly from 84.79 ± 36.84 U/L to 75.26 ± 23.81 U/L (*p* = 0.008).

### 2.6. TG

**Figure 7:**
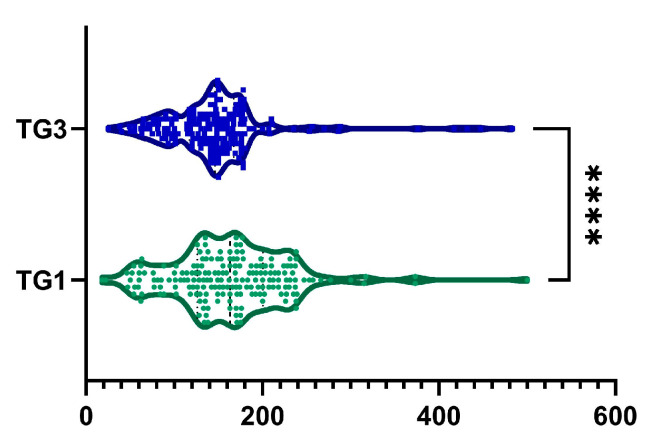
A reduction in TG was observed after 6 months of treatment (TG1, visit I vs. TG3, visit III). ****, *p* < 0.0001.

A 3.18 ± 39.26% reduction in TG levels was observed from baseline to month 6, with a statistically significant difference, as illustrated in Figure 7 (*p*-value < 0.0001). Furthermore, 112 patients (58.9%) demonstrated a reduction in TG levels. Among male patients, we observed a statistically significant decreased TG from 168.8 ± 72.74 mg/dL to 154.89 ± 79.46 mg/dL (*p* = 0.002). Similarly, among female patients, TG decreased statistically from 156.65 ± 57.05 mg/dL to 138.01 ± 39.58 mg/dL (*p* = 0.001).

### 2.7. Total Cholesterol

**Figure 8:**
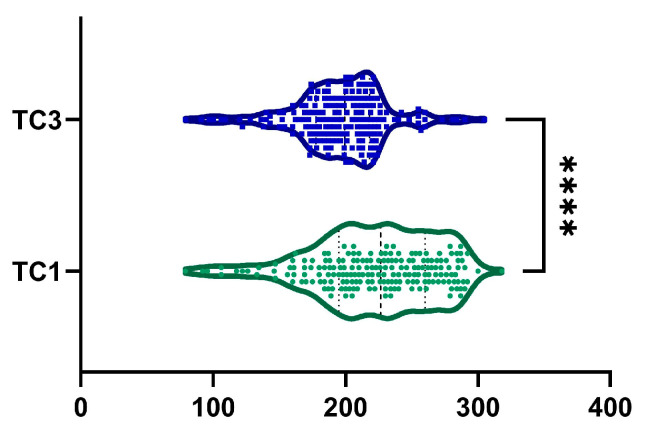
A reduction in TC was observed after 6 months of treatment (TC1, visit I vs. TC3, visit III). ****, *p* < 0.0001.

The decrease in TC levels from baseline to month 6 was 9.51 ± 17.9%, with a statistically significant difference, as shown in Figure 8 (*p*-value < 0.0001). Additionally, 138 patients (72.6%) exhibited a reduction in TC levels. Among male patients, we observed a statistically significant decreased TC from 215.42 ± 47.34 mg/dL to 192.36 ± 34.05 mg/dL (*p* < 0.0001). Similarly, among female patients, TC decreased statistically from 230.84 ± 43.79 mg/dL to 200.69 ± 34.03 mg/dL (*p* < 0.0001).

### 2.8. Blood Glucose

**Figure 9:**
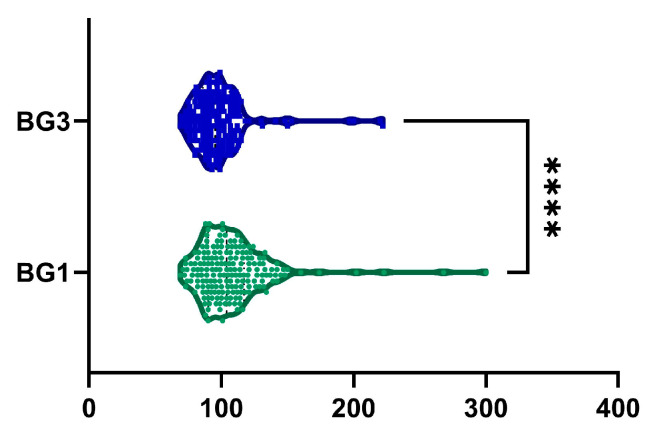
Reduction in blood glucose (BG) was observed after 6 months of treatment (BG1, visit I vs. BG3, visit III). ****, *p* < 0.0001.

The decrease in BG levels from baseline to month 6 was 6.4 ± 20.1%, with a statistically significant difference, as shown in Figure 9 (*p*-value < 0.0001). Additionally, 126 patients (66.3%) exhibited a reduction in BG levels. Among male patients, we observed statistically significant decreased BG levels from 108.6 ± 28.9 mg/dL to 97.9 ± 19.93 mg/dL (*p* < 0.0001). Similarly, among female patients, BG levels decreased statistically from 107.56 ± 28.3 mg/dL to 98.42 ± 23.89 mg/dL (*p* < 0.0001).

### 2.9. Fibrosis Assessment

**Figure 10:**
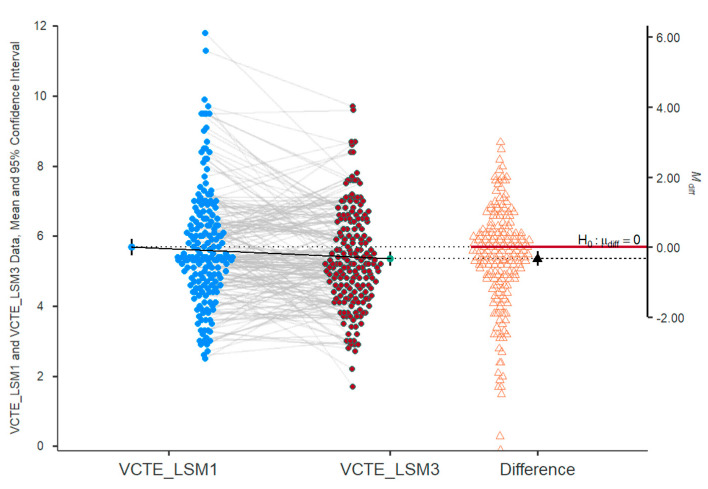
Evolution of fibrosis—VCTE_LSM values from visit I (VCTE_LSM1) to visit III (VCTE_LSM3). The dashed lines represent individual patient trajectories, showing how each patient’s VCTE_LSM value changed over time. The arrow emphasizes the magnitude of the decrease in stiffness (the mean), suggesting an improvement in liver fibrosis over the course of the study.

There was a significant decrease in the VCTE_LSM value from the first visit to the third visit (*p* = 0.002) (Figure 10). An improvement in fibrosis by at least one stage was observed in 27 patients (14.21%). Conversely, the worsening of fibrosis by one stage was observed in 14 patients (7.4%) and by two stages in two patients (1.1%). No differences were observed for 147 patients (77.4%).

### 2.10. Steatosis Assessment

**Figure 11:**
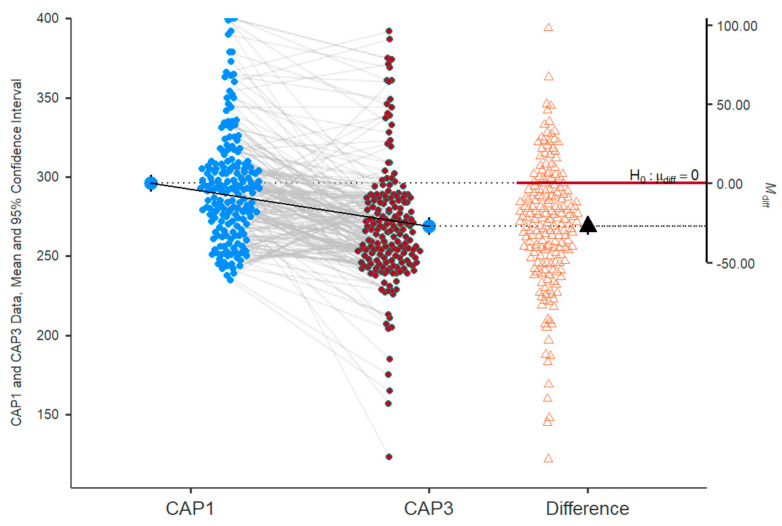
Evolution of steatosis—VCTE_CAP values from visit I (CAP1) to visit III (CAP3). The dashed lines represent individual patient trajectories, showing how each patient’s CAP value changed over time. The arrow emphasizes the magnitude of the decrease in steatosis levels (the mean), suggesting an improvement in liver steatosis over the course of the study. It directly visualizes whether the change was significant and in which direction.

There was a significant decrease in VCTE_CAP from the first visit to the third visit (*p* = 0.002) (Figure 11). An improvement in steatosis by three stages was observed in five patients (2.6%), by two stages in 27 patients (14.2%), and one stage in 72 patients (37.9%). Conversely, the worsening of steatosis by one stage was observed in 18 patients (9.5%) and by two stages in one patient (0.5%). No differences were observed for 67 patients (35.3%).

**Figure 12:**
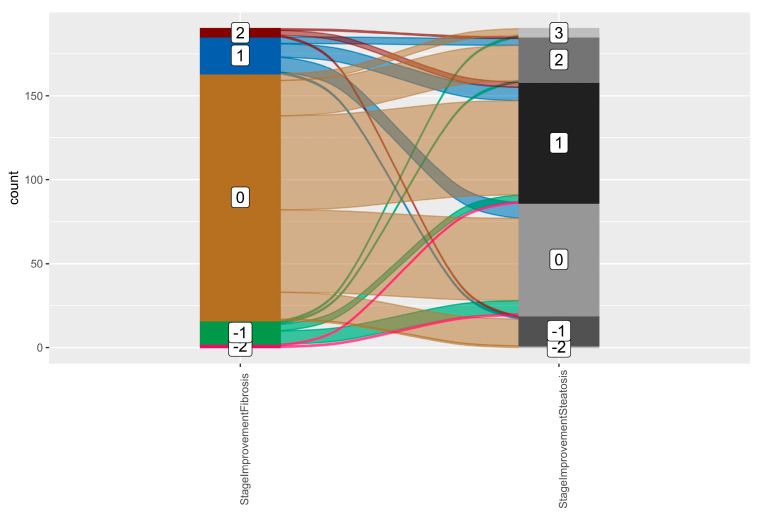
Alluvial diagram to connect fibrosis improvement with steatosis improvement. 1/2/3 = one-/two-/three-stage improvement, 0 = no change in steatosis/fibrosis stage, −1/−2 = worsening by one stage/two stages.

No significant correlation was observed between improvements in fibrosis and steatosis, as illustrated in the alluvial diagram in Figure 12. The width of each stream in the diagram corresponds to the number of subjects within a specific category of fibrosis improvement and steatosis improvement. The flows were evenly distributed without a discernible pattern. Furthermore, Spearman’s rho coefficient was 0.046 (df = 188, *p*-value = 0.53), confirming the absence of a statistically significant correlation.

**Figure 13:**
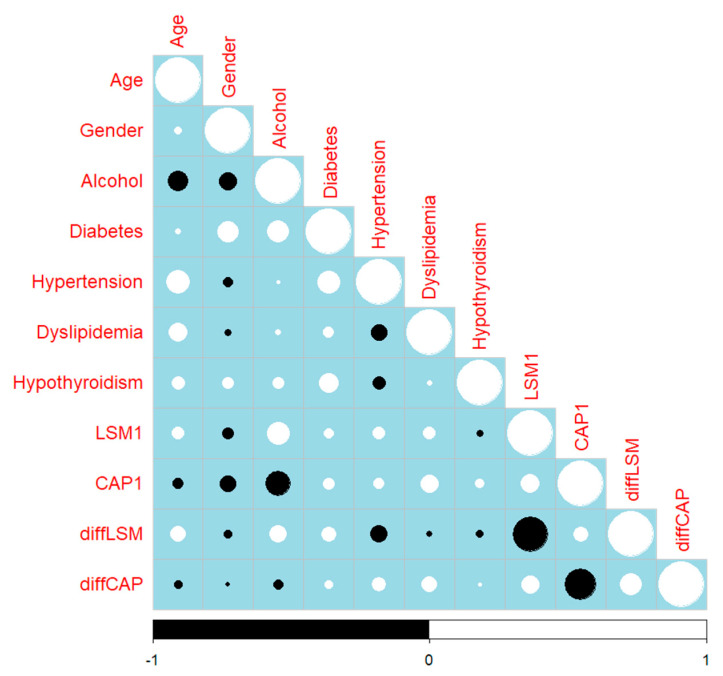
Partial correlation plot. The circles indicate Spearman’s rho coefficient (white color indicates a positive correlation, black color indicates a negative correlation, dimension of the circle indicates the magnitude of the correlation). diffLSM, the difference between liver stiffness measurement (LSM) in visit III and visit I; diffCAP, the difference between controlled attenuation parameter (CAP) in visit III and visit I.

The relationships between baseline characteristics (e.g., age, gender, alcohol consumption, comorbidities such as diabetes, hypertension, dyslipidemia, and hypothyroidism) and changes in LSM and CAP after 6 months of treatment are presented in Figure 13. The differences in LSM (diffLSM) and CAP (diffCAP) were calculated as the change from baseline to the 6-month follow-up. Age did not influence the changes in LSM (rho = 0.026, *p*-value = 725) and CAP (rho = −0.022, *p*-value = 0.766) over the 6-month treatment period. We did not observe any potential impact of gender on the response to treatment, as reflected by changes in LSM (rho = −0.048, *p*-value = 0.508) and CAP (rho = −0.049, *p*-value = 0.503). The role of alcohol consumption in modulating treatment outcomes was assessed, and a patient with no alcohol consumption had significantly better CAP values after 6 months of treatment (rho = 0.143, *p*-value = 0.049). The absence of hypertension was determined to be associated with better changes in LSM (rho = −0.183, *p*-value = 0.012). The analysis revealed a weak but statistically significant positive correlation between the improvement in liver fibrosis (measured by diffLSM) and improvement in liver steatosis (diffCAP) (rho = 0.197, *p*-value = 0.006).

## 3. Discussion

Despite the non-invasive scores proposed for the risk assessment of steatosis, transabdominal ultrasound is the first recommendation for diagnosis of MASLD. A simple score, FIB-4, can decide which patient with MASLD has to be referred for liver fibrosis assessment. A FIB-4 score above 1.3 is ideal for this approach, but our study includes all patients with steatosis diagnosed on the first visit (visit 0) on transabdominal ultrasound and no fibrosis.

In our study, we excluded patients with MASLD and diabetes who received metformin due to the effect on liver enzymes and liver fat content. Despite most studies showing that metformin can reduce hepatic steatosis, there are several reports that indicate otherwise [21,22].

Lifestyle interventions with weight loss in patients with MASLD have shown clear benefits, but adherence to these changes in real life is difficult to adopt. The use of certain medications seems to have a better effect in patients with MASLD and therefore over time, various substances or formulas that act most effectively on the pathophysiological mechanisms have been researched [23]. Since no ideal association has been established, continuous study on the best substance combination formula is mandatory.

Silymarin, essential phospholipids, and vitamin E all offer synergic and additional roles to achieve their benefits in improving liver function. Silymarin, derived from milk thistle, acts as an antioxidant, essential phospholipids are crucial for building and repairing liver cell membranes, and vitamin E, also an antioxidant, protects against free radical damage.

Treatment responses to individual components of our study formula may vary [24]. Pervez et al. reported significant improvements in ALT and AST levels among patients with MASLD. However, these findings contrast with other studies demonstrating no observable effects on ALT levels or histological outcomes in patients receiving vitamin E monotherapy [25].

Trappoliere et al. reported a similar effect of treatment with silymarin, vitamin E, and essential phospholipids on patients with MASLD [26].

These results suggest that the treatment response, as measured by changes in LSM and CAP, is not strongly influenced by baseline demographic or clinical characteristics. This indicates that the treatment may be broadly effective across diverse patient subgroups regardless of age, gender, alcohol consumption, or the presence of common metabolic comorbidities.

The weak positive correlation implies that while there is some degree of association between fibrosis improvements (measured by LSM reduction) and steatosis reduction (measured by CAP decrease), the relationship is not strong. This finding suggests that the mechanisms driving improvements in fibrosis and steatosis may be partially overlapping but are not entirely interdependent. Notably, the treatment demonstrated more pronounced efficacy in ameliorating steatosis compared to its effects on fibrosis regression.

These findings highlight the importance of addressing both fibrosis and steatosis as distinct yet potentially interrelated aspects of liver health in the management of liver disease.

### Limitations

The weak correlation suggests that other factors not accounted for in this analysis may influence changes in LSM and CAP independently. Further research is needed to explore additional variables that could modulate these outcomes, such as specific treatment effects, genetic factors, the length of the treatment (extending the follow-up 6 months), or lifestyle modifications. Extending the treatment duration could provide deeper insights into the sustained efficacy of the intervention (for instance, reductions in liver fat (steatosis) might occur more rapidly than improvements in fibrosis, which typically require a longer duration to manifest). Another limitation of our study was the low number of patients, and a larger cohort can expose more accurate data.

A further study with the aim of comparing the components of the treatment separately and with this formula may demonstrate a clear impact of the benefits of this association.

Another limitation of our study is that the obtained results may not be extrapolated easily to different races due to the fact that all participants were Caucasians, with Romanian nationality.

In our study group, regarding lifestyle, diet, and physical exercise, no specific diet was imposed despite the fact that a Mediterranean diet was suggested. A small group of patients recognized that the diet was modified beneficially but relatively superficially, only over a shorter period than the evaluated one. Therefore, we consider that together with the short follow-up time, the changes determined by the lifestyle change did not have a major impact on MASLD in our study.

## 4. Materials and Methods

We conducted a prospective interventional study for 12 months between January 2024 and December 2024. The study was conducted in 2 centers, in the Research Center of Gastroenterology and Hepatology, University of Medicine and Pharmacy of Craiova, Craiova, Romania, and in the Gastroenterology Department of “Sf. Apostol Andrei” Clinical Emergency County Hospital, Constanta, Romania. The design of the study was to evaluate the response of the patients with MASLD after treatment with supplements of Esentin Trio^®^ containing silymarin (100 mg), vitamin E (150 mg), and essential phospholipids (400 mg). The dosage for the patients accepted in the study was 1 capsule twice daily (BID), morning and evening, as indicated in the medicine’s leaflet, for 3 months or 6 months, depending on the response.

The study was approved by the ethics committee of the University of Medicine and Pharmacy of Craiova, Romania (no. 73/29.01.2024) and by the ethics committee of “Sf. Apostol Andrei” Clinical Emergency County Hospital, Constanta, Romania. All procedures were performed according to the Declaration of Helsinki after signing the informed consent form.

Inclusion criteria:

Adult patients with hepatic steatosis diagnosed on transabdominal ultrasound at the first visit.

Exclusion criteria:

The presence of focal liver lesions;

An alcohol consumption above 20 g/day in women and above 30 g/day in men;

Metformin intake;

Allergy to any of the ingredients of the original formula;

All patients included in the study had to achieve 4 visits (visit 0, visit I, visit II, and visit III) and be registered by a gastroenterologist doctor.

In visit 0, personal data such as name, ID number, age, and address—either urban or rural—were noted. A transabdominal ultrasound using a Logiq E10 scanner (GE HealthCare, Glattbrugg Switzerland) was performed to confirm the presence of liver steatosis and to exclude focal hepatic lesions.

In visit I, the body mass index (BMI), the presence of diabetes, high blood pressure, dyslipidemia, hypothyroidism, alcohol consumption status, and medication recommended at home were noted. In this visit, the patients brought in a recent set of laboratory tests, not older than 7 days, which includes alanine transaminase (ALT), aspartate transaminase (AST), gamma-glutamyl transferase (GGT), alkaline phosphatase (ALP), lipid profile, triglycerides (TGs), total cholesterol (TC), and blood glucose (BG) levels. VCTE was performed by a gastroenterologist, and the results were reported for fibrosis assessment as an LSM (kPa) value and corresponding fibrosis grade, and for steatosis assessment as a CAP (dB/m) value and corresponding steatosis grade. Patients with confirmed steatosis were prescribed 3 months of medication, consisting of 1 Esentin Trio^®^ capsule BID.

During visit II, BMI was noted. A transabdominal ultrasound was performed to assess the resolution or persistence of hepatic steatosis. Patients with persistent steatosis were prescribed an additional 3 months of medication, in the same posology, 1 Esentin Trio^®^ capsule BID.

At visit III, BMI was noted. Patients were required to provide a fresh set of blood test results (≤ 1 week old) measuring AST, ALT, GGT, ALP, TG, TC, and BG. VCTE was performed by a gastroenterologist. The results were reported for steatosis assessment as a CAP (dB/m) value with a corresponding steatosis grade and for fibrosis assessment as an LSM (kPa) value with corresponding fibrosis grade.

The interval of fibrosis and steatosis was decided using the recommended values of Fibroscan (Echosens, France): Steatosis grades intervals: S0: 100–233.5 dB/m; S1: 233.6–268.5 dB/m; S2: 268.6–300.9 dB/m; S3: 301 dB−400 dB/m.

The degrees of fibrosis were defined using the following values recommended by Fibroscan (Echosens, Paris, France): F0–F1: 1–6.9 kPa; F2: 7–8.6 kPa; F3: 8.7–10.2 kPa; F4: 10.3–75 kPa. All investigations were performed by a gastroenterologist with expertise in abdominal ultrasound and VTE. No specific diet was required during this study despite a Mediterranean diet being suggested.

Normal intervals of laboratory tests value ranges: AST 2–34 U/L, ALT 3–44 U/L, GGT 0–55 U/L, ALP 50–116 U/L, TG 0–150 mg/dL, TC 70–240 mg/dL, and BG 70–110 mg/dL.

All data was entered into an online platform developed by the Department of Medical Informatics and Biostatistics, University of Medicine and Pharmacy of Craiova, Romania, which allowed us to export of the entire database of the study for forward statistical examination. We used R packages retrieved from CRAN, https://cran.r-project.org (easyalluvial, seolmatrix, qgraph) and GraphPad PRISM 10.4.1 software, and statistical significance was set to 0.05. Continuous variables were expressed as the mean ± standard deviation (SD) or median (interquartile range, IQR) depending on the distribution of the data. We performed the Kolmogorov–Smirnov test to compare the empirical distribution of our data to a normal distribution. Categorical variables were summarized as frequencies and percentages. Changes in liver stiffness (diffLSM) and steatosis (diffCAP) were calculated as the difference between baseline and 6-month follow-up values. We used a paired statistical Wilcoxon test to determine if there are statistically significant differences after 6 months of treatment. Spearman’s rank correlation coefficient (rho) was used to assess the relationship between changes in liver stiffness (diffLSM) and steatosis (diffCAP) and baseline characteristics, as the data were not normally distributed. The correlation between improvements in fibrosis stages and steatosis stages were illustrated in the alluvial diagram, where the width of each stream corresponds to the number of subjects within specific categories, observing a clear pattern.

Regarding the sample size, given the lack of prior data on this triple combination, we adopted a pragmatic approach to ensure feasibility while achieving statistical power for primary endpoints. We included a post hoc power analysis using G*Power 3.1.9.7. The power test was calculated assuming an alpha level of 0.05, and the patients from both baseline and sixth-month groups yielded a power level (1-β) that ranged from 99.4% to 100% for our analysis.

## 5. Conclusions

From baseline, visit 0, to month 6th, visit III, a statistically significant difference reduction was observed in ALT, AST, GGT, ALP, TG, total cholesterol, and blood glucose levels. Additionally, fibrosis values demonstrated a significant decrease from the first visit to the last visit, with 14.21% of cases showing improvement by at least one stage. Similarly, steatosis values decreased significantly, with improvements distributed as follows: three-stage reduction in 2.6% of cases, two-stage in 14.2% of cases, and one-stage reduction in 37.9% of cases.

Even though administered separately, silymarin, essential phospholipids, and vitamin E have established their efficacy in MASLD; this study demonstrates that their combination induces an indubitable effect on liver steatosis, even in a short period of time, of 6 months, and it can be proposed due to it having no adverse effects on patients with MASLD.

## Figures and Tables

**Table 1:** Demographic and baseline characteristics.

Characteristics	Esentin Trio^®^ (*n* = 190)
Age, years	
Mean (SD)	47.66 ± 10.43
Median (Q1, Q3)	47 (39.75–55.25)
Sex, male, *n* (%)	100 (52.6%)
BMI, kg·m^−2^	
Mean (SD)	28.97 ± 4.24
Median (Q1, Q3)	29 (26–32)
Alcohol consumption, *n* (%)	
No	50 (26.3%)
Occasional	109 (57.4%)
Frequent	25 (13.2%)
Daily	6 (3.2%)
Type 2 diabetes, *n* (%)	67 (35.3%)
Hypertension, *n* (%)	77 (40.5%)
Dyslipidemia, *n* (%)	94 (49.5%)
Hypothyroidism, *n* (%)	29 (15.3%)
Fibrosis grade, *n* (%)	
F0–F1	156 (82.1%)
F2	23 (12.1%)
F3	10 (5.3%)
F4	1 (0.5%)
Steatosis grade, *n* (%)	
S1	42 (22.1%)
S2	74 (38.9%)
S3	74 (38.9%)

**Table 2:** Comparison between clinical characteristics at baseline and 6 months.

Clinical Characteristics	Baseline	After 6 Months of Treatment	*p*-Value
FibroScan VCTE/LSM, kPa			0.018
mean (SD)	6.14 ± 6.68	5 ± 1.42	
median (Q1, Q3)	5.4 (4.6–6.5)	5 (4.4–6.4)	
FibroScan CAP, dBm			<0.0001
mean (SD)	296.05 ± 36.82	269 ± 39.22	
median (Q1, Q3)	293 (271.5–310)	266 (246.25–286.75)	
ALT, UI^−1^			<0.0001
mean (SD)	58.65 ± 38.44	37 ± 20.68	
median (Q1, Q3)	46.5 (27.75–84.25)	30 (20–49.75)	
AST, UI^−1^			<0.0001
mean (SD)	49.5 ± 31.07	33 ± 16.6	
median (Q1, Q3)	40.5 (23–69)	28 (20.3–43)	
GGT, UI^−1^			<0.0001
mean (SD)	73.47 ± 48.54	57 ± 35.96	
median (Q1, Q3)	59 (34–115)	45 (28–86)	
ALP, UI^−1^			0.022
mean (SD)	79.33 ± 33.03	74 ± 24.68	
median (Q1, Q3)	75.5 (57.83–95.5)	70 (58–88.75)	
TG, mg·dL^−1^			<0.0001
mean (SD)	163.04 ± 65.89	147 ± 64	
median (Q1, Q3)	163 (126–200.5)	146 (117–167)	
TC, mg·dL^−1^			<0.0001
mean (SD)	222.73 ± 46.22	196 ± 35.26	
median (Q1, Q3)	226.5 (195–260)	200 (178–217.75)	
BG, mg/dL			<0.0001
mean (SD)	108.11 ± 28.55	98 ± 21.84	
median (Q1, Q3)	104 (90–117.25)	96 (86–105.75)	

BMI, body mass index; VCTE/LSM, vibration-controlled transient elastography–liver stiffness measurement; ALT, alanine transaminase; AST, aspartate transaminase; GGT, gamma-glutamyl transferase; ALP, alkaline phosphatase; TG, triglyceride; TC, total cholesterol; BG, blood glucose, SD, standard deviation, Q1, first quartile; Q3, third quartile.

## Data Availability

The original contributions presented in this study are included in the article. Further inquiries can be directed to the corresponding author.

## Abbreviations

The following abbreviations are used in this manuscript:

MASLD	Metabolic dysfunction-associated steatotic liver disease
MASH	Metabolic dysfunction-associated steatohepatitis
MRE	Magnetic resonance elastography
VCTE	Vibration-controlled transient elastography
CAP	Controlled attenuation parameter
LSM	Liver stiffness measurement
ROS	Reactive oxygen species
LDL	Low-density lipoproteins
TG	Triglycerides
MG	MILLIGRAMS
BID	Twice daily
ID	Identity document
BMI	Body mass index
ALT	Alanine transaminase
AST	Aspartate transaminase
GGT	Gamma-glutamyl transferase
ALP	Alkaline phosphatase
TC	Total cholesterol
BG	Blood glucose
CAP	Controlled attenuation parameter
SD	Standard deviation
Q1	First quartile
Q3	Third quartile
